# MAPK/ERK Signaling in Osteosarcomas, Ewing Sarcomas and Chondrosarcomas: Therapeutic Implications and Future Directions

**DOI:** 10.1155/2012/404810

**Published:** 2012-04-12

**Authors:** Chandhanarat Chandhanayingyong, Yuhree Kim, J. Robert Staples, Cody Hahn, Francis Youngin Lee

**Affiliations:** ^1^Center for Orthopedic Research (COR), Department of Orthopedic Surgery, Columbia University, New York, NY 10032, USA; ^2^Department of Orthopedic Surgery, Faculty of Medicine Siriraj Hospital, Mahidol University, Bangkok 10700, Thailand

## Abstract

The introduction of cytotoxic chemotherapeutic drugs in the 1970's improved the survival rate of patients with bone sarcomas and allowed limb salvage surgeries. However, since the turn of the century, survival data has plateaued for a subset of metastatic, nonresponding osteo, and/or Ewing sarcomas. In addition, most high-grade chondrosarcoma does not respond to current chemotherapy. With an increased understanding of molecular pathways governing oncogenesis, modern targeted therapy regimens may enhance the efficacy of current therapeutic modalities. Mitogen-Activated Protein Kinases (MAPK)/Extracellular-Signal-Regulated Kinases (ERK) are key regulators of oncogenic phenotypes such as proliferation, invasion, angiogenesis, and inflammatory responses; which are the hallmarks of cancer. Consequently, MAPK/ERK inhibitors have emerged as promising therapeutic targets for certain types of cancers, but there have been sparse reports in bone sarcomas. Scattered papers suggest that MAPK targeting inhibits proliferation, local invasiveness, metastasis, and drug resistance in bone sarcomas. A recent clinical trial showed some clinical benefits in patients with unresectable or metastatic osteosarcomas following MAPK/ERK targeting therapy. Despite *in vitro* proof of therapeutic concept, there are no sufficient *in vivo* or clinical data available for Ewing sarcomas or chondrosarcomas. Further experimental and clinical trials are awaited in order to bring MAPK targeting into a clinical arena.

## 1. Introduction

Prior to the era of classical cytotoxic chemotherapeutic agents in the 1970's, patients with osteosarcomas or Ewing sarcomas rarely survived 10 years even after imputative tumor resection [1]. The introduction of cytotoxic chemotherapeutic agents such as doxorubicin or methotrexate was a paradigm shift in oncology practice in the 1970's and 1980's [2]. Chemotherapy allowed limb salvage operations and prolonged survival in 60–80% of patients suffering from osteosarcomas and Ewing sarcomas [3, 4]. Despite this initial leap in sarcoma patient care, about 20–40% of patients with nonresponding, unresectable, or metastatic sarcomas desperately wait for alternative ways of eliminating their cancer.

Additionally, chondrosarcoma patients compose of a group that relies on treatment via surgical resection of primary and metastatic lesions [5, 6]. Many chondrosarcomas arise in the axial skeletons which provide anatomic complexities for wide surgical excisions. As a result, there has been a common notion that chondrosarcomas do not respond to chemo- and radiotherapy.

Despite an increase in knowledge with regards to sarcomas, survival time has not increased over the past 20 years. However, strong waves of new therapeutic opportunities have emerged into the sarcoma field by targeting several key pathways governing oncogenesis and aggressive clinical features. One such avenue of targeting is the MAPK/ERK kinase pathway that relays upstream oncogenic signals from the Ras/Raf, IGF, EGF, PDGF, and VEGF to downstream effectors of cancer-related gene expression [7, 8]. This review paper is intended to highlight an emerging role of MAPK/ERK targeting with respect to the three most common bone sarcomas.

## 2. MAPK Signaling in Cancers

### 2.1. General Perspectives

Cancer cells have sustainable and self-sufficient machinery for uncontrolled growth. “The Hallmarks of Cancer” by Hanahan and Weinberg describe six unusual characteristics of cancer in comparison to normal cells [15]. They include sustaining proliferative signaling, evading growth suppressors, resisting apoptosis, enabling replicative immortality, inducing angiogenesis, and activating invasion and metastasis. Recently, Hanahan and Weinberg proposed 4 additional hallmarks of cancer such as an abnormal energy metabolism, the evasion of the immune system, chromosomal abnormalities with genetic diversity, and inflammation [16]. Inflammation itself consists of a series of cellular and molecular events that overlap with the other hallmarks of cancer.

**Figure 1:**
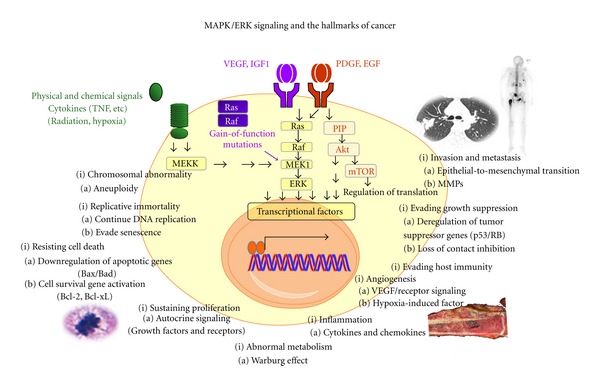
MAPK/ERK signaling and the hallmarks of cancers. The MAPK/ERK pathway mediates several upstream signals from well-known oncogenic growth factors and proinflammatory stimulants. Activation of the MAPK/ERK pathway by growth factors, proinflammatory stimulants and gain-of-function mutations of Ras/Raf promotes phenotypic changes characteristic of cancer cells [9–14].

Extracellular Receptor Kinase (ERK) proteins are a family of protein-serine/threonine kinases that are activated via the phosphorylation of tyrosine in response to growth factors such as insulin and nerve growth factor (NGF). ERK is also known as the Mitogen-activated Protein Kinase (MAPK), and plays a major role in mediating inflammatory as well as oncogenic signals. MAPK is activated by *M*APK/*E*RK *K*inase (*MEK*). Ras/Raf is upstream of MEK. In the classical setting, MEK is activated by many upstream growth factors/cytokine receptors in response to radiation, hypoxia, physical forces, TNF, RANKL, and TLR. When gain-of-function mutations occur in Ras/Raf, a commonly observed phenomenon in many types of cancers, MEK/MAPK proteins become constitutively activated. MAPK/ERK signaling fulfills many cancer hallmarks by the mediation of mitosis and stem-cell-ness, production of matrix degrading enzymes, Warburg effect, angiogenesis, bone destruction, cytokine production, chromosomal aberration, and anergy [9–13] (Figure 1).

Well-known growth factor receptor like IGF, EGF, VEGF, and PDGF activate the MAPK/ERK pathway. Various other receptors including TLR, TNFRF, and PTH also participate in its activation. These receptor signaling pathways regulate gene expression for cytokines, chemokines, growth factors, cell proliferation, and antiapoptosis. Interestingly, these major receptor pathways all cross at the MAPK/ERK similar to Penn Station or Charles de Galle Airport where many trains or airplanes (i.e., signals) converge. Convergence of these cellular signals makes the MAPK/ERK pathway an attractive and powerful therapeutic target given that this pathways inhibition could disarm several hallmarks of cancer at a single convergence point (Figure 1). This concept has been tested recently in experimental and clinical settings.

The expression and activation of the MAPK pathway correlate with prognosis and influences therapeutic outcome in several types of cancer. Expression of pRaf (ser 338), an upstream activator of MEK1, was associated with disease relapse and decreased overall survival of patients with breast cancer who were treated with tamoxifen. Thus, MAPK/ERK activation may play a role in antiapoptosis and Tamoxifen resistance [17]. Patients with metastatic gastric cancers expressing p-MAPK/ERK in tumor showed decreased disease-free overall survival (8.5 months) in comparison to nonexpressing cancers (13.7 months) indicating p-MAPK as a negative prognostic indicator in metastatic gastric cancers [18]. In glioblastoma, heightened p-MAPK expression was associated with poor response to radiotherapy and worse overall survival rate [19]. Targeting the MAPK/ERK pathway in hepatocellular carcinomas, thyroid, hepatobiliary, lung, and solid cancers using selumetinib (AZD 6244), a MAPK 1/2 inhibitor, has shown promising results [20–22].

## 3. MAPK Signaling in Bone Sarcomas

**Figure 2:**
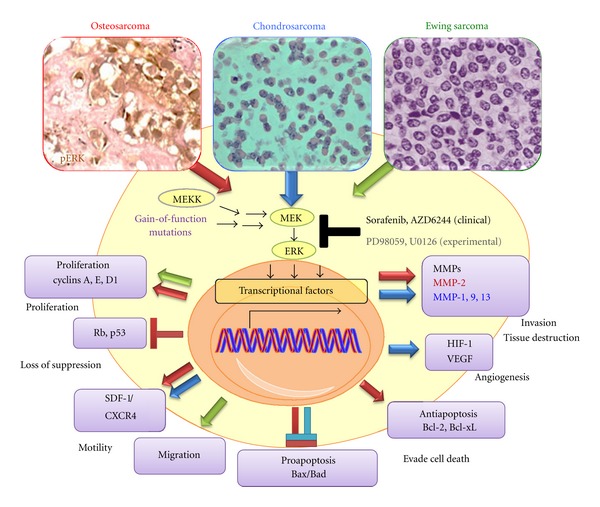
MAPK/ERK signaling in osteosarcomas, Ewing sarcomas, and chondrosarcomas.

There is a relative paucity of literature concerning the role of MAPK/ERK in bone sarcomas. Various viewpoints about the significance of MAPK/ERK demonstrate that this pathway's role in sarcoma is still being unraveled. It is known that osteosarcomas, Ewing sarcomas, and high-grade chondrosarcomas exhibit heightened pMAPK/pERK1/2 expression (Figure 2). Therefore, targeting MAPK/ERK1/2 could develop a modern molecular adjuvant therapy for bone sarcomas. To understand the therapeutic importance of RAF-MEK-ERK pathway targeting in bone sarcomas, it is necessary to update the results of experimental and clinical trial data.

## 4. Osteosarcoma

### 4.1. Limitations of Current Therapy

Osteosarcoma is the most common primary malignant bone tumor in adolescent and young adults and accounts for approximately 20% of all bone malignancies [23]. Current treatment involves a multidisciplinary approach with surgery and chemotherapy; however, 20–40% percent of patients does not respond to conventional treatment and have a dismal 5-year survival rate. Recent studies have shown that overexpression and abnormal activation of Raf/MEK/ERK signaling pathway may regulate tumor proliferation, migration, and metastasis in osteosarcoma as well as in other malignancies [24–26].

### 4.2. *In Vitro * Data

While most studies suggest that the inhibition of ERK 1/2 leads to increased apoptosis and decreased metastasis [24–26], some studies conclude that the activation of RAF/MAPK/MEK/ERK1/2 pathway is required for osteosarcoma cells apoptosis [27]. Targeted inhibition of EGFR, one of the upstream signals, in five osteosarcoma cell lines reduced motility, colony formation, and invasiveness; whereas inhibitors of Her-2, nerve growth factor receptor (NGF-R), and PDGF receptor (PDGF-R) had no effect [28]. The study by Noh et al. [24] examined the therapeutic effect of PD98059 (an inhibitor of ERK1/2 phosphorylation) on osteosarcoma cell lines *in vitro *(Figure 2). PD 98059 increased the expression of proapoptotic proteins such as Bax and induced cell death. Doxorubicin paradoxically upregulated antiapoptotic proteins such as Bcl-2 and Bcl-xL by osteosarcoma cells *in vitro*. ERK1/2 inhibition prevented doxorubicin-induced upregulation of Bcl-2 and Bcl-xL, thereby increasing doxorubicin sensitivity in osteosarcoma cells. In human osteosarcoma cell line SaOS-2 cells, MEK/MAPK is a negative regulator of differentiation while p38 MAPK promotes differentiation [29]. Interaction between stromal cell-derived factor-1 (SDF-1) and its receptor (CXCR-4) increases motility of osteosarcoma cells through a pERK pathway [30]. pERK inhibitor PD98059 inhibited motility of human osteosarcoma cells *in vitro*, suggesting that pERK inhibitors impede local invasion and metastasis.

### 4.3. *In Vivo * Data

Various targeted inhibitors have been shown to have antitumor effects in osteosarcoma (Table 1). An *in vivo* survival study using 143B human osteosarcoma cells with elevated Ras activity demonstrated that pERK targeting with PD98059 resulted in slower tumor growth and prolonged survival by inducing the production of proapoptotic proteins. Combinatorial treatment with doxorubicin and PD98059 further prolonged the survival of osteosarcoma-bearing mice. These data suggest a potential benefit of using MAPK/ERK inhibitors as a molecular adjuvant agent in addition to conventional cytotoxic drugs (Table 1). Some osteosarcoma cell lines express that functional insulin-like growth factor 1 receptor (IGF-1R) on their cell surface that, in turn, stimulates proliferation. The therapeutic efficacy of pharmacologic inhibitors of the IGF-1R pathways has been explored in bone sarcomas. IGF-1R inhibition with monoclonal antibodies resulted in growth retardation and prolonged event-free survival in osteosarcoma-bearing mice [31–33]. Sorafenib, a small molecule Raf kinase and vascular endothelial growth factor (VEGF) receptor kinase inhibitor, which is upstream of MAPK, is approved by FDA for treatment of renal cell carcinomas and hepatocellular carcinomas [34]. Sorafenib treatment caused reduction in tumor volume and lung metastasis in osteosarcoma xenograft [35].

### 4.4. Clinical Trials

In osteosarcoma, expression of VEGF-R3 is associated with poor event-free and overall survival while VEGF-B correlated with a poor histological response to chemotherapy [42]. Apparently, there was no statistically significant correlation between clinical outcome and expression of MAPK in osteosarcoma [42]. However, immunohistochemical expression analyses have inherent limitations of quantification in archived specimens. A Phase I study with Sunitinib, a multitargeted tyrosine kinase inhibitor of signaling downstream of VEGFR, PDGFR, FLT-3, B-Raf, and c-Kit, showed no objective responses in bone sarcomas except one “stable disease” response out of 2 patients with osteosarcoma [43] (Table 1). Another multicenter Phase II trial has shown promising effects of Sorafenib in 35 patients with relapsed and unresectable high-grade osteosarcoma following conventional cytotoxic agents [37]. The median overall survival was 7 months. Sixteen patients (46%) were free from disease progression after 4 months of therapy. The overall response rate was 49%. 5 patients (14%) showed partial (>30% shrinkage in its widest diameter) or minor response (<30%). 10 out of 35 (29%) patients showed the clinical benefit of partial and minor response as well as stable disease. Of 4 patients with stable disease, PET scan showed decreased FDG uptake in the area of osteosarcoma indicating cell death within the lesion. There were no patients that showed complete response suggesting that combinatorial treatments either with other targeting agents or conventional cytotoxic drugs are necessary for better outcomes.

## 5. Ewing Sarcoma

### 5.1. Limitations of Current Therapy

Ewing sarcoma is the second most common primary bone tumor occurring in children and young adults. Treatment consists of chemotherapy followed by wide resection and may include radiation. It is considered to have the worst prognosis in bone sarcoma. EWS is characterized by the t(11; 22)(q24; q12) translocation resulting in EWS-ETS fusion gene, which presents as EWS-FLI1 in over 85% of cases [44, 45]. EWS/ETS fusions function as transcriptional regulators that can activate downstream gene expression.

### 5.2. *In Vitro * Data

Oncogenic function of EWS-FLI1 depends on IGF-1 signaling [46]. Ewing sarcoma cells consistently express IGF-1R and insulin receptor (IR) [47, 48]. IGF-1R-mediated pathway appears as a major autocrine loop in pathogenesis and maintains cell functions for EWS [49]. The EWS-FLI1 requires the presence of IGF-1R in order to transform murine fibroblasts [50]. Two major downstream IGF pathways, that are, MAPK and PI3K, play an important role in Ewing sarcoma. Activation of mTOR, ERK and NF-kB was found in Ewing sarcoma tumor cell lines [51]. PD98059 and U0126 (MEK inhibitor) impaired Ewing sarcoma cell growth by inducing G1 blockage and reducing its migratory effect [47] (Figure 2). Another study confirmed that combined administration of U0126 and LY294002 (PI3K inhibitor) enhanced actinomycin-D-induced apoptosis *in vitro* and *in vivo* [38]. ERK1/2 proteins are constitutively activated in transformed NIH 3T3 cells expressing EWS/FLI-1 and ERK inhibition impaired the ability of EWS/FLI-1 overexpression to transform NIH3T3 [48]. IGF receptor targeting alone may not be effective since Ewing sarcoma cells can switch to alternative signaling pathways from IGF-1R to IR-A to maintain sustained activation of ERK1/2. It is strategically more advantageous to target MAPK/ERK at a converging point downstream of growth factor receptors.

### 5.3. *In Vivo * Data

*In vivo* studies are carried out to assess the effect of IGF-1R blocker on bone tumor (Table 1). CP-751,871, a human IGF-1R blocker, showed significant growth inhibition in Ewing sarcoma-bearing mice. Combinatorial treatment with rapamycin showed synergistic effects [52]. NVP-AEW541, a dual pan-PI3K-mTOR inhibitor, inhibited growth of Ewing sarcoma and metastasis in athymic mice [53]. There was paradoxical activation of pERK most likely due to activation of alternative pathways following mTOR inhibition. Sorafenib resulted in tumor growth inhibition in Ewing sarcoma *in vivo* model [36].

### 5.4. Clinical Trials

The outcome of a Phase I trial on Sorafenib-treated patients with Ewing sarcoma is in progress and the results have not yet been reported [39]. IGF-1R receptor signaling is linked to MAPK/ERK. Clinical trials in patients with Ewing sarcoma showed approximately 10–15% response rate following single-agent treatment with IGF-1R antibody targeting EWS-FLI1 or EWS-ERG [54]. In phase I trials, IGF-1R inhibitors (R1507, AMG479, CP-751, 871) showed sustained remission in patients with Ewing sarcoma [33, 55, 56]. In a phase II clinical trial, Imatinib Mesylate targeting multiple kinases including MAPK/ERK showed only one partial response among 24 patients with Ewing sarcoma [57]. Resistance eventually develops from monotargeted therapy because cancer cells utilize alternative pathways. Many efforts try to downfall resistance by combining two or more targeted inhibitors. The combined regimen consisting of IGF-1R and mTOR inhibitors demonstrated good responses in two patients with refractory EWS after administration of IGF-1R inhibitors [58].

## 6. Chondrosarcoma

### 6.1. Limitations of Current Therapy

The current mainstay of treatment is wide resection. However, many chondrosarcomas arising in the axial skeleton are not amenable to wide resection [5, 6]. Extensive Grade 1 chondrosarcomas in the pelvis and spine can lead to mortality due to the destruction or obstruction of key vital anatomic structures such as the aorta, vena cava, intestines, kidneys, and liver. Higher grade (grade 2 or 3) and dedifferentiated chondrosarcomas are associated with pulmonary metastasis and death [5]. Chemotherapy has been largely ineffective for high-grade or unresectable chondrosarcoma due to the presence of inhomogeneous vascularity, low pH and increased interstitial pressure, p-glycoprotein, and activation of cell survival pathways all of which potentially impede drug delivery and pharmacologic action [5, 6].

### 6.2. *In Vitro * Data

Low-grade chondrosarcoma cells often resemble normal chondrocytes in terms of type II collagen and matrix synthesis. Low-grade chondrosarcomas are avascular. They also share ihh/PTHrP signaling pathways which tightly control chondrocytic differentiation. Grade II or III chondrosarcomas lose chondrocytic phenotypes and have more vascularization. Chondrosarcomas are notorious for their unusual resistance to radiation and chemotherapy [5, 6]. Chondrosarcoma cells use growth factor signaling for enhanced growth [59]. Sorafenib induced dose- and time-dependent inhibition of pERK and apoptosis in two different chondrosarcoma cell lines [60]. Sorafenib also inhibited the expression of cyclin D1, Rb, and antiapoptotic proteins Bcl-xL. MMP-1 is commonly expressed in locally invasive chondrosarcomas (Figure 2). Hypoxic condition results in the upregulation of MMP-1 by chondrosarcoma cells. pERK inhibition by siRNA prevented hypoxia-induced MMP-1 upregulation [61, 62]. Osteopontin is a bone matrix protein that increases the migration and expression of matrix metalloproteinase (MMP)-9 in grade II human chondrosarcoma cells through pERK1/2 pathway [63]. Both PD98059 and U0126 inhibited osteopontin-induced MMP-9 upregulation and migration of human chondrosarcoma cells. Integrins are a family of transmembrane-binding proteins that bidirectionally activate cell signaling pathway with the extracellular matrix (ECM) to promote tumor cell proliferation, differentiation, and migration. Integrin alpha (v) beta3 (*α*v*β*3) has been shown to be highly correlated with bony metastasis in multiple cancers [64, 65]. Integrin *α*v*β*3 is highly expressed in chondrocytes and is upregulated on chondrosarcoma cell migration [66]. Integrin *α*v*β*3 is regulated by activation of transforming growth factor beta 1 (TGF-*β*1) and bone morphogenic protein 2 (BMP-2), and the process depends on PI3K and MEK/ERK signaling [67, 68]. One study showed that chondrosarcoma cell migration is induced by TGF-*β*1 and BMP-2 through CCN and integrin *α*v*β*3 expression leading to activation of FAK/MEK/ERK pathway. *α*5*β*1 monoclonal antibody and MEK inhibitors (PD98059 and U0126) inhibited migration of chondrosarcoma cells [69].

### 6.3. *In Vivo * Data

There does not seem to be any *in vivo* data on the use of MAPK/pERK targeting in chondrosarcomas *in vivo* at the present time.

### 6.4. Clinical Trials

 pERK may have a diagnostic value in grading chondrosarcomas. Results of immunohistochemical staining of pERK in pathologic specimens of 45 chondrosarcomas and 21 enchondromas showed more augmented pERK expression in higher grade chondrosarcomas [70]. There are no large chondrosarcoma series which provide meaningful insights into the value of MAPK/ERK targeting. Available clinical trials were conducted using a mixed bag of sarcoma cases (Table 1). A Phase II trial of sorafenib showed prolonged stable disease for duration of 37 weeks in one patient with chondrosarcoma [40]. Another phase II study of sorafenib in patients with recurrent or metastatic sarcoma indicates prolonged stable disease over 6 months in 2 chondrosarcoma patients [41].

## 7. Future Direction

Targeted therapy for sarcomas is still at an early stage. *In vitro* proof of therapeutic concept studies assures that pMAPK/ERK targeting offers new methods of inhibiting proliferation, invasion, angiogenesis, and inflammation which are the hallmarks of cancers. *In vivo* experiments successfully demonstrated the inhibition of tumor growth and prolonged survival of osteosarcoma-bearing mice. Recent Phase II clinical trial data showed a clinical benefit rate of 30% in patients with relapsed and unresectable high-grade osteosarcomas following MAPK/ERK targeting therapy. There is a knowledge gap concerning the efficacy of MAPK/ERK targeting for Ewing sarcomas or chondrosarcomas *in vivo* or in a clinical setting. Available studies also suggest that a single-agent-targeted therapy may not provide clinically meaningful anticancer effects. Specific MAPK/ERK inhibitors are emerging as additional adjuvant repertoires for multimodality therapy. Further *in vivo* experiments are needed to provide stronger rationale for initiating clinical trials. More collaborative multicenter clinical trials are necessary to recruit a sufficient number of patients and to draw meaningful conclusions on the efficacy of combinatorial cytotoxic agents and modern molecular adjuvant therapy.

## Figures and Tables

**Table 1:** Therapeutic implications of Ras-Raf-MEK-ERK targeting in bone sarcomas.

Sarcoma types	Study design	Target	Inhibitor types	Results	References
	In vivo*	pERK1/2	MEK inhibitor (PD98059)	Prolonged survival increase chemosensitivity	[24]
Osteosarcoma	In vivo*	pERK1/2	RAF inhibitors (Sorafenib)	Growth inhibition	[36]
	In vivo*	pERK1/2	RAF inhibitors (Sorafenib)	Decrease lung metastasis antitumoral activity	[35]
	Clinical Trial (Phase II) (*N* = 35)	pERK1/2	RAF inhibitors (Sorafenib)	Clinical benefit (PR + MR + SD) >6 months = 29%	[37]
	In vivo*	pERK1/2	RAF inhibitors (Sorafenib)	Growth inhibition	[36]
Ewing's sarcoma	In vivo*	pERK1/2 PIP3K	U0126 LY294002	Increase chemosensitivity	[38]
	Clinical trial (Phase I) (*N* = 34)	pERK1/2	RAF inhibitors (Sorafenib)	Not reported (ongoing)	[39]
Chondrosarcoma	Clinical trial (Phase II) (*N* = 26)	pERK1/2	RAF inhibitors (Sorafenib)	Prolonged stable disease	[40]
	Clinical trial (Phase II) (*N* = 147)	pERK1/2	RAF inhibitors (Sorafenib)	Prolonged stable disease for >6 months	[41]

PR: Partial response; MR: Minor response; SD: Stable disease, *Human sarcoma xenografts in mice.
